# Supplementary material for: Learning curve and surgical outcome of robotic assisted colorectal surgery with ERAS program
Source: Sci Rep. 2022 Nov 29;12:20566. doi: 10.1038/s41598-022-24665-w (PMC9709162; doi:10.1038/s41598-022-24665-w)
Supplement: Supplementary file 2 — Supplementary Information 2. [file 41598_2022_24665_MOESM2_ESM.zip › RAL Raw data-Ñ[▒K/049/Patho_ñw╝╨░O▒Kñσ.pdf]

臺中榮民總醫院 臺中市臺灣大道四段1650號 病理部活體組織切片 臨床醫學實驗及病理診斷中心 檢驗(查)報告

[redacted]

Pathologic diagnosis:
1. Sigmoid colon, robotic anterior resection --- Adenocarcinoma, moderately differentiated. Tumor invaded to muscularis propria. The bilateral cut ends and mesocolic lymph nodes (0/16) are free of tumor.
2. Colon, cut end, distal, resection --- Unremarkable.
3. IMA root, dissection --- Unremarkable (0/1).
4. Jejunum, resection --- Gastrointestinal stromal tumor of low risk. The resection margins are free of tumor.

Ancillary study for diagnosis:
1. IHC stains for MLH1, MSH2, PMS2 and MSH6 are done on section F. All above markers are preserved.
2. CD117 (+) IHC stain done on section Q.

Prognostic and predictive factor:
(for colon cancer)
1. Tumor size: 1x1 cm.
2. Depth of invasion: Muscularis propria.
3. Histologic grade: G2: Moderately differentiated.
4. Angiolymphatic invasion: Not identified.
5. Perineural invasion: Not identified.
6. Tumor deposits (discontinuous extramural extension): Not identified.
7. Macroscopic tumor perforation: Not identified.
8. Surgical Margins: a) Proximal Margin: Uninvolved by invasive carcinoma, distance of tumor from margin: 6 cm. b) Distal Margin: Uninvolved by invasive carcinoma, distance of tumor from margin: 3 cm. c) Radial or Mesenteric Margin: Uninvolved by invasive carcinoma, distance of tumor from margin: >2 cm.
9. Lymph node status: Uninvolved (0/17).
10. Extranodal involvement: Not identified.
11. Tumor regression grading S/P CCRT: No known presurgical therapy.
12. Type of polyp in which invasive carcinoma arose: Not identified.
13. Additional pathologic findings: Not identified.
14. Pathological TNM Stage: pT2N0(According to the eighth edition, American Joint Committee on Cancer Staging Guidelines for Tumors).
15. TNM descriptors: Not applicable.

Prognostic and predictive factor:
(for GIST)
1. Tumor site: Specify: Jejunum.
2. Tumor size: 25x25 mm.
3. Tumor focality: Unifocal.
4. GIST subtype: Spindle cell.
5. Mitotic rate: <1/ 5 mm2 (approximately 20 to 25 HPF. See Note).
6. Necrosis: Not identified.
7. Histologic grade: G1: Low grade; mitotic rate ≦5/ 5 mm2.
8. Margins: Uninvolved by GIST, distance of tumor from closest margin (cm): 1 cm, specify margin: circumferential.
9. Risk assessment: Low risk.
10. Regional Lymph Nodes: No lymph nodes submitted or found.
11. Distant metastasis: Cannot be assessed.
12. Prebiopsy treatment (select all that apply): No therapy.
13. Treatment effect: No known presurgical therapy.
14. Pathological TNM stage: pT2Nx (According to the eighth edition, American Joint Committee on Cancer Staging Guidelines for Tumors).
15. TNM descriptors: Not applicable.

Gross description:
The specimen consists of 1) a segment of colon, 10 cm in length and 4 cm in proximal cut end circumference, 5 cm in distal circumference, and mesocolic soft tissue. A gray tan firm annular elevated and centrally ulcerative tumor, 1x1 cm, is noted, 3 cm to the distal cut end. On cross section, the tumor invades to the muscle layer with more than 20 mm to the radial margin. The both cut ends are free of tumor grossly. 2) distal cut end, a piece of tan mucosa-covered soft tissue, 2x1.5x0.8 cm. 3) IMA root, a piece of tan yellow soft tissue, 4x3x0.8 cm. 4) a segment of jejunum, 10 cm in length and 6 cm in circumference. There is a gray white firm transmural tumor, 2.5x2.5 cm, with 3 cm to the nearest cut end. Representative part for section: A) proximal cut end B) distal cut end C) circumferential margin D-G) tumor H) colon J) specimen 2 K) specimen 3 L-M) cut ends of jejunum N-R) tumor of jejunum S) mesetery soft tissue T) mesocolic lymph nodes.

Note:
1. The interpretation of MMR panel and recommended studies are as below: (1) No loss of nuclear expression of MMR proteins: low probability of microsatellite instability-high (MSI-H). (2) Loss of nuclear expression of MLH1 and PMS2: testing for methylation of the MLH1 promoter and/or mutation of BRAF is indicated (the presence of a BRAF V600E mutation and/or MLH1 methylation suggests that the tumor is sporadic and germline evaluation is probably not indicated; absence of both MLH1 methylation and of BRAF V600E mutation suggests the possibility of Lynch syndrome, and sequencing and/or large deletion/duplication testing of germline MLH1 may be indicated). (3) Loss of nuclear expression of MSH2 and MSH6: high probability of Lynch syndrome (sequencing and/or large deletion/duplication testing of germline MSH2 may be indicated, and, if negative, sequencing and/or large deletion/duplication testing of germline MSH6 may be indicated). (4) Loss of nuclear expression of MSH6 only: high probability of Lynch syndrome (sequencing and/or large deletion/duplication testing of germline MSH6 may be indicated). (5) Loss of nuclear expression of PMS2 only: high probability of Lynch syndrome (sequencing and/or large deletion/duplication testing of germline PMS2 may be indicated). (6) Please note: There are exceptions to the above IHC interpretations. These results should not be considered in isolation, and clinical correlation with genetic counseling is recommended to assess the need for germline testing.
2. The type of specimen fixation and processing: Formalin-fixed paraffin-embedded sections.
3. The antibody clone(vendor) and detection system used: CD117: polyclonal rabbit(DAKO)/VENTANA, CD34: monoclonal mouse(DAKO)/VENTANA. Type of method: LSAB.
4. The test is fixed in 10% neutral buffered formalin for at least 6 hours and no longer 24 hours.
5. The required total count of mitoses is per 5 mm2 on the glass slide section. With the use of older model microscopes, 50 HPF is equivalent to 5 mm2. Most modern microscopes with wider 40X lenses/fields require approximately 20 to 25 HPF to encompass 5 mm2.

#T-59300_2 #M-81403_2 2 1105 M0000F
#T-C4560_2 #M-09183_2 0 1105 000000
#T-58402_2 #R-1030A_2 9 1104 S00000

已電子簽章

敬告：本單僅供醫師參考使用，不具任何申請效力！！
請病患妥為保管，若不慎遺失需自負個人資料外洩責任！
